# Medical Convention

**Published:** 1847-04

**Authors:** 


### MEDICAL CONVENTION.

From all that now appears, the Medical Convention in Philadelphia, which is to meet on the first Wednesday in May, will be large. The medical schools and societies in all the States are appointing delegates, so that the convention may be expected to reflect the sentiments of the profession in every part of the country. A multitude of projects will of course be brought forward, some of them Utopian enough, but some, it may be hoped, of a wise and salutary tendency. Where such contrariety must prevail in the sentiments of the members, forbearance, moderation and a spirit of concession will be necessary to harmonious action and ultimate good. Professor Paine, of New York, may be considered as heading one party likely to have a voice in the convention. If some are too revolutionary in their views, Prof. P. is ultra conservative. He has proposed his plan of medical reform. We append it without note or comment:

"Notwithstanding I am among those who believe that medical education is carried to a much greater extent in this country than in any other as it respects the mass of the profession, and believe also that the American physician surpasses the European in philosophical views of disease and in practical habits, there is no one who can desire more than myself to see the standard of excellence still further advanced. For this purpose, only one experimental plan has presented itself to my mind as at all likely to result auspiciously, and it is one which cannot fail of being decisive.

"It is assumed by many that any existing school which may extend its term of instruction, and exact a higher preliminary education, will find its reward in an increased number of students. Upon this basis, which appears to be urged by the advocates of 'reform' as a motive to the nonconformists, I would respectfully propose that the summary step be taken by the national convention of physicians, which is soon to meet in Philadelphia, to institute a college upon the contemplated plan of 'reform,'—embracing of course in its objects a requisition of extended preliminary acquirements, a multiplication and extension of lecture terms, an examination of the candidates for graduation by other hands than their teachers, etc.

"If the desired 'reform' can be obtained, it surely must be most feasible under the united concurrence and influence of the whole profession; and the moment a successful demonstration is made, all other colleges must necessarily, as it appears to me, adopt the example, however it may curtail the number of educated physicians."—*Boston Medical and Surgical Journal.*

